# Antimicrobial pharmacokinetics and stewardship in critically Ill adult patients receiving ECMO: Challenges, evidence, and future directions

**DOI:** 10.1016/j.jhlto.2025.100438

**Published:** 2025-11-13

**Authors:** A.L. Dzierba, Y-H. Liang, H. Lyster

**Affiliations:** a Department of Medicine, New York University Grossman School of Medicine, New York, NY; b Department of Pharmacy, University Hospitals Bristol and Weston NHS Foundation Trust, Bristol, UK; c Department of Pharmacy, Royal Brompton & Harefield Hospitals, part of Guy’s & St Thomas’ hospital, London, UK; d Department of Cardiothoracic Transplantation and Mechanical Support, Harefield Hospital, Royal Brompton & Harefield Hospitals, Part of Guy’s and St. Thomas’ NHS Foundation Trust & Imperial College, London UB9 6JH, UK; e Institute of Pharmaceutical Science, School of Cancer & Pharmaceutical Sciences, King’s College London, London, UK

**Keywords:** antibacterials, antifungals, antivirals, pharmacokinetics, pharmacodynamics, antimicrobial dosing, extracorporeal membrane oxygenation

## Abstract

Infection is a common indication and complication for extracorporeal membrane oxygenation (ECMO), and adult ECMO recipients face increased nosocomial infection risk. Effective antimicrobial dosing in these patients remains difficult due to pharmacokinetic (PK) and pharmacodynamic (PD) alterations driven by critical illness and extracorporeal circuits. In this narrative review we aim to discuss the potential impact of ECMO on antimicrobial PK and dosing requirements. Findings across studies show heterogeneity in dosing practices and indicate that inter-patient variability is influenced more by critical illness and organ dysfunction than ECMO alone. Standardized dosing protocols remain lacking, and in the absence of robust guidelines, current best practice involves applying PK/PD principles, using therapeutic drug monitoring, and individualizing dosing strategies. Across all antimicrobial drug classes, robust prospective studies linking PK/PD targets to clinical outcomes are lacking. Future research should focus on prospective trials correlating dosing regimens with meaningful clinical endpoints to refine evidence-based antimicrobial guidance. These efforts are essential to developing evidence-based dosing recommendations and optimizing antimicrobial stewardship in this high-risk population.

## Introduction

Infection is a common indication for extracorporeal membrane oxygenation (ECMO) initiation, such as in cases of acute respiratory distress syndrome secondary to bacterial or viral pneumonia, and continues to be a frequent complication during ECMO support. Adult patients requiring ECMO support have been independently associated with an increased risk of nosocomial infections, with reported prevalence rates ranging from 9% to 65%.^1^ Therefore, effective antimicrobial dosing is a critical aspect of care. Determining the optimal antimicrobial dosing strategy in ECMO-supported patients remains challenging, in part because clinical response is often assessed using surrogate markers that may be nonspecific or unreliable indicators of infection resolution. Furthermore, significant pharmacokinetic and pharmacodynamic (PK/PD) alterations resulting from both critical illness and the ECMO circuit can lead to unpredictable drug concentrations causing treatment failure or increased risk of toxicity.^2^

Timely initiation of appropriate antimicrobial therapy is critical to the successful treatment of infections. Contemporary guidelines increasingly support PK/PD-driven dosing strategies to maximize antimicrobial effectiveness.^3^ Accordingly, optimizing antimicrobial therapy in ECMO-supported critically ill patients requires a comprehensive understanding of the PK disturbances stemming from both critical illness and extracorporeal support.

### PKPD concepts and considerations

**Figure 1:**
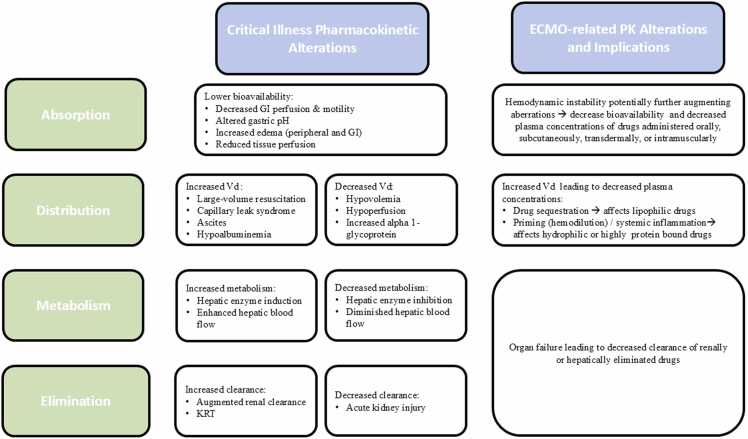
Pharmacokinetic changes during critical illness and ECMO-support.^5^, ^102^, ^103^

Pharmacokinetics describes the time-dependent movement of a drug through the body, including absorption, distribution, metabolism, and excretion.^4^ In critically ill patients, PK is often profoundly altered due to dynamic changes in protein binding, fluid shifts, decreased perfusion, and impaired drug clearance, all of which can significantly affect drug exposure and therapeutic outcomes (Figure 1).^5^ Organ support systems, while essential for stabilizing critically ill patients and enabling recovery or intervention, may contribute to additional physiologic derangements. Among these, ECMO and continuous renal replacement therapy (CRRT) are particularly impactful modalities that further modify drug PK, necessitating individualized dosing strategies. As such, a clear understanding of the patient’s critical illness trajectory—along with the initiation, duration, and cessation of supportive devices—is vital to guide dosing adjustments and minimize the risk of therapeutic failure or toxicity. Premature dose reduction during episodes of transient acute kidney injury risk early antibiotic efficacy, potentially increasing the risk of mortality. This concern is particularly pronounced in patients with sepsis, where achieving adequate antimicrobial exposure within the first 48 h is critical for optimal clinical outcomes.^6^, ^7^

At both initiation and during ECMO support, drug sequestration is promoted by contact with circuit components—particularly the membrane oxygenator and polyvinyl chloride (PVC) tubing—that offer substantial surface area for adsorption.^2^, ^8^ Knowledge of a drug’s physicochemical characteristics can help anticipate the extent and clinical relevance of sequestration. Drugs that are highly lipophilic or exhibit strong protein binding (>80–90%) are particularly susceptible to sequestration within the ECMO circuit.^9^, ^10^ Lipophilicity is often quantified using the octanol-water partition coefficient (log P), with values ≥ 2.0 indicating a higher likelihood of partitioning into organic materials such as PVC tubing, resulting in reduced circulating drug concentrations.^11^ In contrast, hydrophilic drugs may exhibit an increased volume of distribution during ECMO, influenced by circuit-related factors such as the addition of priming solutions (e.g., plasma, crystalloids, or albumin) and the activation of an inflammatory response leading to capillary leak, particularly early in the ECMO course. Drug sequestration can also vary based on circuit age, the type of membrane oxygenator, and the chemical composition and length of the tubing. Non-pulsatile cardiac support devices can activate the renin-angiotensin system, leading to increased circulating volume and reduced renal clearance, further complicating drug elimination.^12^, ^13^

Pharmacodynamics describes the relationship between drug concentration and bacterial killing or growth inhibition, typically referenced against the minimum inhibitory concentration (MIC)—the lowest concentration that prevents bacterial growth in vitro for antimicrobials.^14^ Only the free, unbound portion of a drug is active against pathogens. Factors like high protein binding and expanded volume of distribution may limit tissue penetration, potentially leading to therapeutic failure despite sufficient plasma concentrations.^15^

Pharmacodynamic indices that correlate with antimicrobial efficacy include: (I) the duration that free drug concentrations remain above the MIC (fT>MIC), (II) the ratio of maximum concentration (C_max_) to MIC, and (III) the ratio of total drug exposure over 24 h, measured as the area under the concentration-time curve (AUC₀_–_₂₄), to MIC.^16^ Time-dependent agents, such as beta-lactams, require sustained fT>MIC to achieve optimal bacterial killing. In contrast, concentration-dependent antimicrobials—such as aminoglycosides and fluoroquinolones—exert their maximal effect when peak concentrations reach 8 to 10 times the MIC (C_max_/MIC). Other agents, such as glycopeptides, demonstrate mixed time- and concentration-dependent properties, with their efficacy best predicted by the AUC₀_-_₂₄/MIC ratio.

Given the aforementioned considerations, PK/PD aberrations limit the applicability of dosing regimens derived from non-critically ill populations. Relying on such extrapolations may lead to subtherapeutic exposure or drug toxicity. Tailoring therapy to the PK/PD changes associated with critical illness is essential to maximize efficacy and reduce the risk of adverse effects.

### Critical analysis of existing literature

Table 1, Table 2 summarize the potential PK changes in critically ill patients on ECMO and dosing recommendations for antibacterial, antifungal and antiviral agents.

**Table 1:** Potential PK Changes in Critically Ill Patients on ECMO for Antibacterial Agents

Drug	Log P	Protein Binding (%)	PKPD Index	Expected ECMO Sequestration Effect	Dosing Guidance
Beta-lactams					
Cefepime	–4 to –0.4	∼20	% fT>MIC	Minimal circuit loss Vd: increased	▪ Dosing similar to critically ill not on ECMO ▪ Standard dose 2 g q8-12h ▪ Extended infusion may help with high MIC ▪ TDM if available due to poor target attainment
Ceftazidime-avibactam	<−1/<−1	<10%/ <10%	% fT>MIC	Minimal circuit loss	▪ Standard dose
Cefiderocol	<1	40−60%	% fT>MIC	Minimal to moderate	▪ Standard dose
Ceftriaxone	–1.7	85−95	% fT>MIC	Moderate to significant circuit loss Vd: increased	▪ Higher end of standard dose ▪ 2 g q12-24h
Ceftolozane- tazobactam	<−1/<−1	16−21%/ 30%	% fT>MIC	Minimal circuit loss	▪ Standard dose
Meropenem	–0.6	∼2	% fT>MIC	Minimal circuit loss Vd: increased	▪ Dosing similar to critically ill not on ECMO ▪ TDM-guided dosing if available due to poor target attainment ▪ Higher end of the standard dose (3-6g/d). ▪ Use extended (over 3-4h) or continuous infusions with TDM
Piperacillin-tazobactam	0.5/−2.0	20−40	% fT>MIC	Minimal circuit loss Vd: increased	▪ Dosing similar to critically ill not on ECMO ▪ Mixed data ▪ 4.5 g q6h ▪ Use extended or continuous infusions with TDM due to poor target attainment
Aminoglycosides					
Gentamicin, Tobramycin, Amikacin	<0	<30	AUC_0–24_/MIC C_max_/MIC	Minimal circuit loss Vd: increased CL: decreased	▪ Dosing similar to critically ill not on ECMO ▪ TDM guided dosing
Cyclic lipopeptides					
Daptomycin	–5.1	84−93	AUC_0–24_/MIC	Moderate circuit loss	▪ Limited data. ▪ 10−12mg/kg/d for the first 48 h to cover higher MIC then adjust according to renal function.
Glycopeptides					
Vancomycin	–2.6	50	AUC_0–24_/MIC	Minimal circuit loss Vd: increased	▪ LD 20−30mg/kg. ▪ MD 15−20mg/kg q8-12h or continuous infusion with TDM
Teicoplanin	0.5	90	AUC_0–24_/MIC	Moderate circuit loss	▪ Higher doses, especially the first 14 days ▪ LD 12mg/kg for 3−5 doses. Then guided by renal function and TDM if available
Oxazolidinones					
Linezolid	0.7−0.9	31	AUC_0–24_/MIC	Minimal circuit loss Vd: increased	▪ Mixed data. ▪ 600 mg q8h monitor for toxicity, or use alternative agents ▪ Use TDM if available
Macrolides					
Azithromycin	3−4	7−51	AUC_0–24_/MIC	Minimal circuit loss Vd: increased	▪ Limited data ▪ Standard dose
Others					
Co-trimoxazole (sulfamethoxazole-trimethoprim)	0.9	70/44	Unclear	Minimal to moderate circuit loss	▪ Limited data. ▪ Standard dose with TDM.

**Table 2:** Potential PK changes in critically ill patients on ECMO for antifungal and antiviral agents

Drug	Log P	Protein Binding (%)	PKPD Index	Expected ECMO Sequestration Effect	Dosing Guidance
AZOLES					
Fluconazole	0.56	12	AUC_0–24_/MIC	▪ Minimal circuit loss ▪ ^V^d^:^ increased	▪ Insufficient adult data ▪ May require increased LD
Voriconazole	2.56	58	AUC_0–24_/MIC	▪ Moderate to significant circuit loss	▪ Conflicting data ▪ Dosing similar to critically ill not on ECMO ▪ TDM-guided dosing
Posaconazole	5.5	>98	AUC_0–24_/MIC	▪ Moderate to significant circuit loss	▪ TDM-guided dosing
Isavuconazole (active moiety)	3.6	>98	AUC_0–24_/MIC	▪ Moderate to significant circuit loss	▪ TDM-guided dosing
ECHINOCANDINS					
Anidulafungin	1.87	>99	AUC_0–24_/MIC	▪ Moderate circuit loss ▪ ^V^d^:^ increased	▪ Insufficient adult data ▪ Dosing similar to critically ill not on ECMO
Caspofungin	−2.8	97	AUC_0–24_/MIC	▪ Moderate circuit loss ▪ ^V^d^:^ increased	▪ Mixed data ▪ A higher-than-standard empirical dosing regimen (e.g., a loading dose of 100 mg on day 1, followed by a maintenance dose of 100 mg daily)
Micafungin	0.4	>99	AUC_0–24_/MIC	▪ Moderate circuit loss ▪ ^V^d^:^ increased	▪ Contradictory data ▪ Dosing similar to critically ill not on ECMO
POLYENES					
Amphotericin deoxycholate	0.8	>90	C_max_/MIC	▪ minimal	▪ Standard dosing, monitor adverse effects
Liposomal amphotericin	highly lipohilic	>90	C_max_/MIC	▪ moderate	▪ Conflicting data, consider increased dosing
ANTIVIRAL AGENTS					
Ganciclovir	−2.5	1−2	AUC_0–24_/MIC	▪ Minimal circuit loss ▪ ^V^d^:^ increased	▪ Insufficient data ▪ Use standard doses with TDM
Oseltamivir	1.1	42	Unclear	▪ Minimal circuit loss	▪ Use standard doses
Remdesivir (prodrug – active metabolite)	1.9	90	Unclear	▪ Moderate circuit loss ▪ ^V^d^:^ increased	▪ Use standard doses

**Table 3:** Key Messages/recommendations

Key Messages
• There is a lack of established dosing protocols for antimicrobials in ECMO-supported critically ill patients • ECMO circuits can alter antimicrobial pharmacokinetics, but critical illness itself is often a more significant contributor to variability • Empirical dosing should account for drug properties – lipophilic, protein-bound drugs are more prone to ECMO circuit sequestration • Delaying renal dose adjustment in transient acute kidney injury during the first 48 h of therapy for wide therapeutic index antimicrobials • Dosing should be optimized using therapeutic drug monitoring to avoid therapeutic failure as well as drug accumulation and toxicity • Use of extended/continuous infusions for beta lactams (in particular if no access to TDM) • High-quality, prospective studies are urgently needed to link antimicrobial exposure with clinical outcomes and guide evidence-based dosing

### Antibacterials

As previously mentioned, beta-lactams such as ceftriaxone, meropenem and piperacillin-tazobactam are time-dependent antibacterials. They are cornerstone in the treatment of life-threatening bacterial infections in critically ill patients due to their broad spectrum efficacy and favorable safety profile.^17^, ^18^ Studies have demonstrated poor target attainment in both ECMO and non-ECMO supported patients, which is associated with increased intensive care unit length of stay.^12^, ^19^, ^20^, ^21^, ^22^, ^23^, ^24^, ^25^, ^26^ To ensure adequate coverage of higher MICs, particularly in the setting of altered PK, the higher end of standard dosing ranges, extended or continuous infusions where feasible are advisable, irrespective of renal function, in the initial or early phase of treatment.^27^, ^28^ Therapeutic drug monitoring (TDM), where available, has been suggested to optimize therapy – enhancing efficacy in patients with augmented renal clearance and reducing the risk of toxicity in those with renal impairment.^29^, ^30^

PK data on daptomycin in ECMO settings are limited, although available evidence indicates that ECMO does not significantly affect its disposition.^31^, ^32^, ^33^, ^34^ Zhang et al demonstrated that creatinine clearance (CrCL) affects daptomycin PK while ECMO had no significant effect, suggesting a dosing regimen of 10–12mg/kg depending on CrCL.^31^ As such, dosing strategies reported in the literature appear to rely primarily on CrCL, rather than on ECMO status.

Within the glycopeptides class, poor target attainment of vancomycin have been reported in patients on ECMO,^19^, ^35^ however, several studies have reported similar PK parameters between ECMO and non-ECMO supported patients.^12^ Teicoplanin appears to differ in this regard. Evidence suggests that a dosing regimen of 6 mg/kg may be insufficient for achieving therapeutic targets in patients receiving ECMO, including those on CRRT, particularly in the early stages of therapy.^36^ Various dosing regimens have been proposed across studies with comparable sample sizes.^37^, ^38^ Teicoplanin should be dosed using a weight-based loading dose, followed by maintenance dosing guided by TDM.^37^

Poor target attainment of linezolid has been reported more frequently in patients receiving ECMO compared to non-ECMO controls.^24^ A case series indicated that standard dosing of 600 mg every 12 h was generally sufficient only for pathogens with MICs ≤1 mg/L. Based on limited available data, an increased dose of 600 mg every 8 h, should be used to treat severe infections.^28^ TDM is advisable when available due to substantial intra-patient and inter-patient PK variability.^10^

Data on the use of azithromycin during ECMO are sparse. Existing PK evidence suggests that ECMO does not significantly alter the PK of azithromycin.^39^

A case report by Dhanani et al. described the use of co-trimoxazole at a dose of 100mg/20mg/kg/day for the treatment of *Pneumocystis jirovecii* pneumonia, with no significant PK alterations observed in the context of ECMO.^40^

Several prospective observational studies have investigated the PK of amikacin in patients receiving ECMO. Two studies found no significant impact of ECMO on amikacin PK and recommended dosing strategies consistent with those used in non-ECMO patients.^41^, ^42^ In contrast, Touchard et al and the PHARMECMO study reported suboptimal target attainment in 39% and 33.3% of patients respectively receiving amikacin at 25 mg/kg, suggesting potential variability and the need for individualized TDM in this population.^23^, ^43^ Another prospective population PK study reported that ECMO had only a secondary influence on amikacin PK compared to the impact of renal impairment. The authors recommended a dose of 40 mg/kg in patients with normal renal function, and 25 mg/kg in those with severe renal impairment.^44^ Data on gentamicin and tobramycin remain limited, with existing evidence primarily derived from studies in neonates, infants, in vitro experiments, and animal models.^45^, ^46^, ^47^, ^48^

Current data on use of the newer antibacterials, while limited, indicate that for ceftazidime-avibactam usual dosing could be used in ECMO patients, with target attainment observed in most cases, renal clearance was found to be the main factor to influence ceftazidime and avibactam concentrations.^49^ An ex vivo ECMO model demonstrated similar findings for ceftazidime.^50^ With regards to ceftolozane-tazobactam similarly no dose adjustments were required.^51^, ^52^

There was no sequestration of cefidericol in an ex vivo ECMO circuit,^53^ and in a small case series of five patients, the authors concluded that no dose adjustments were needed, with current recommended dosing based on renal function achieving 100% target attainment for susceptible bacteria.^54^

### Antifungals

In adult patients receiving ECMO, PK studies of antifungal agents reveal variable impacts of extracorporeal support on drug disposition, largely influenced by each agent's physicochemical properties such as lipophilicity and protein binding.

Fluconazole being hydrophilic with low protein binding undergoes minimal circuit sequestration,^9^, ^55^ though an increased volume of distribution may justify a larger loading dose in critically ill patients.^27^, ^56^, ^57^, ^58^ The other azole antifungals are more lipophilic and protein bound, making them more susceptible to circuit-related losses. Voriconazole exhibits moderate lipophilicity and protein binding which could lead to potential circuit sequestration.^59^ Clinical studies results are inconsistent, hence, TDM is recommended to guide dosing.^60^, ^61^ Posaconazole and isavuconazole, both highly lipophilic and extensively protein-bound are at significant risk of ECMO sequestration. While ex-vivo evidence suggests significant posaconazole sequestration^62^ one prospective multicentre clinical study (in six patients) reported no meaningful impact of ECMO on its PK, supporting standard dosing while recommending TDM.^63^ Case reports show conflicting data for Isavuconazole^64^, ^65^, ^66^, ^67^ A study in 7 patients on ECMO determined that while isavuconazole concentrations might be influenced by the higher volume of distribution due to ECMO therapy, large inter-patient variability were observed, they were not altered by the ECMO oxygenator.^64^

While the echinocandins, anidulafungin, caspofungin, and micafungin, have varying lipophilicity characteristics, they all exhibit very high protein binding which raises concerns of circuit sequestration in patients receiving ECMO. Caspofungin is the most studied, often having conflicting results; a case series involving lung transplant recipients receiving caspofungin reported no significant differences in PK parameters between patients on ECMO (n=12) and those not on ECMO (n=7).^68^ Similarly, the ASAP ECMO study, which evaluated nine critically ill patients receiving caspofungin during ECMO support, demonstrated PK profiles comparable to those previously described in critically ill patients not undergoing ECMO.^19^ However, the population PK of the ASAP ECMO study patients concluded that higher-than-standard empirical dosing regimen (e.g., a loading dose of 100 mg on day 1, followed by a maintenance dose of 100 mg daily) is likely advantageous for critically ill patients receiving ECMO.^69^ Other clinical studies have observed the effect of ECMO on the PK of the other echinocandins, anidulafungin and micafungin, to be minimal.^70^, ^71^

Amphotericin B is highly protein bound, and in its conventional form (deoxycholate) is hydrophilic, while the liposomal formulation (LAmB) is highly lipophilic, raising concerns about sequestration in the ECMO circuit. While limited, existing case data show that conventional amphotericin B at standard doses (1 mg/kg) can achieve therapeutic levels in ECMO patients.^72^ Case reports on the use of LAmB for treating invasive aspergillosis in patients receiving ECMO suggest that PK remain unaffected, with drug concentrations staying within the therapeutic range^73^ and PK parameters comparable to those observed in critically ill patients not on ECMO.^74^ However, contrasting reports have documented substantial drug loss to the ECMO circuit. In one case, this necessitated an increased LAmB dose of 10 mg/kg daily,^66^ while another report described a switch to conventional amphotericin B deoxycholate.^75^ Both cases involved adult patients on ECMO being treated for blastomycosis, these inconsistent findings, underscore the need for further clinical research and consideration for higher dosing of LAmB.

### Antivirals

Pharmacokinetic studies of ganciclovir reveal significant inter- and intra-individual variability, while standard intravenous ganciclovir dosing at 5mg/kg every 12 h is generally adequate in adult patients supported with ECMO, case reports have suggested TDM may be necessary to prevent adverse reactions and account for intra-individual variability.^76^, ^77^

Pharmacokinetic studies of oseltamivir (and its active metabolite, oseltamivir carboxylate) in adult ECMO patients demonstrate minimal circuit-related losses. A prospective study of 14 H1N1 adults on VV-ECMO receiving 75 mg every 12 h found oseltamivir carboxylate systemic exposures equivalent to historical controls.^78^ Although CRRT substantially increases clearance, ECMO alone did not necessitate dose adjustment in patients with preserved renal function.^79^

In the case of remdesivir, a prodrug rapidly metabolized to its active nucleoside analog, ECMO may reduce plasma levels due to circuit adsorption,^80^, ^81^ though clinical significance appears minimal. In a cohort of nine critically ill COVID-19 patients (5 on VV ECMO support), standard regimens (200 mg loading, 100 mg daily) achieved mean remdesivir and GS-441524 exposure comparable to non-ECMO data.^82^

Data on antiretroviral (ART) and direct-acting antiviral (DAA) drugs in ECMO patients are sparse. A case report of ARTs, ritonavir, darunavir, tenofovir, and lamivudine indicate that most ARTs reach expected plasma concentrations following dosing, though lamivudine may exhibit unpredictable PK changes requiring individualized monitoring.^83^ For hepatitis C DAAs, no significant ECMO-related PK alterations have been reported, but data are limited and TDM is recommended when feasible.

## Management recommendations

### Big picture approach to dose optimization – guide

**Figure 2:**
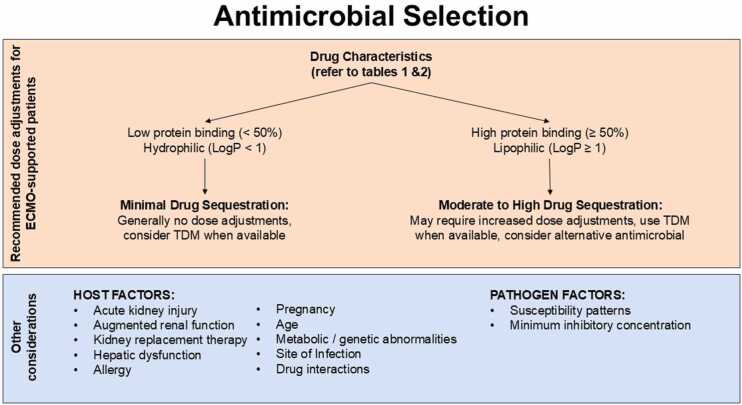
Antimicrobial dosing considerations in ECMO-supported patients.

Dosing antimicrobials in ECMO-supported, critically ill patients is challenging. ECMO-related PK/PD research remains limited and key dosing questions continue to lack definitive recommendations. Most published studies on PK variability are limited by being single-center with small patient cohorts, reliance on ex-vivo models, and inconsistent consideration of concomitant extracorporeal supports (e.g., ECMO with CRRT); additionally, heterogeneity in patient-specific factors such as BMI and genetics may influence results. However, in the absence of robust data, antimicrobial dosing in ECMO-supported patients should be guided by patient-specific factors in combination with the drug’s physicochemical properties (Figure 2).

The PK of antimicrobials are largely comparable to those reported in critically ill patients not receiving ECMO support^19^; however, some nuances may apply. Drugs with moderate to high sequestration in the ECMO circuit may require dose escalation due to an increased volume of distribution (Vd). In contrast, hydrophilic drugs typically do not require ECMO-specific dose adjustments. However, the PK of hydrophilic antimicrobials can still be significantly altered by critical illness itself. Therefore, clinicians often initiate certain hydrophilic agents, such as beta-lactams, at the higher end of the dosing range during the early phase of infection to ensure target attainment, particularly given their wide therapeutic index. For drugs with a narrow therapeutic index, such as aminoglycosides, vancomycin, and polymyxins, delayed dose adjustment, particularly in the setting of kidney impairment, poses an unacceptable risk of treatment-related toxicity.

### Challenges / recommendations

There is a paucity of ECMO-related PK/PD research, with existing studies marked by notable limitations. Many studies describing the impact of the ECMO circuits on PK variability are limited by single-center designs, small sample sizes, and frequent exclusion of other extracorporeal circuits (e.g., ECMO with CRRT). Furthermore, many studies use in vitro models that offer valuable insights into drug sequestration within ECMO circuits, but do not adequately capture the clinical realities of critically ill patients. (e.g., drug metabolism or drug clearance) that may further influence PK. These models also lack integration of meaningful PD targets, which are essential for guiding and optimizing antimicrobial therapy in ECMO-supported patients.

Timely therapeutic drug monitoring is essential to ensure optimal antimicrobial target attainment in ECMO-supported patients; additionally, regular use of TDM-guided dosing where available is important e.g. triazole antifungals where dynamic adjustments may be required.^84^ While beta-lactam antimicrobials are the most widely prescribed antimicrobials in the ICU, the routine use of beta-lactam TDM is limited to a select number of centers worldwide; where TDM is not readily available, a more viable option would be to infuse beta-lactams as a prolonged or continuous infusion to optimize target attainment which can significantly improve time above MIC (T>MIC)—the primary PD driver of efficacy for these agents. By prolonging the infusion duration, we can better maintain plasma concentrations above the MIC throughout the dosing interval, even in patients with fluctuating renal function.^85^, ^86^, ^87^ Ultimately, while TDM remains ideal, strategic dosing approaches like extended infusion and deferred adjustment can help bridge the gap in real-world practice where TDM is limited.

ECMO-supported patients represent a highly heterogeneous population. While PK/PD target attainment is important across all indications, heightened vigilance is warranted in patients with active infection, who may exhibit greater PK variability^88^, ^89^ than those receiving antimicrobials for prophylaxis.

### Antimicrobial stewardship

Antimicrobial stewardship programs are essential in the care of critically ill patients where infection-related morbidity and mortality are high, and antimicrobial use is often empiric, broad-spectrum, and prolonged. It involves a multidisciplinary approach aimed at optimizing antimicrobial use, minimizing patient harm, and limiting the spread of drug resistance.^90^, ^91^ There are several key challenges magnified in adult patients receiving ECMO support.

Antimicrobial stewardship in adult patients receiving ECMO remains a complex and evolving field.^92^ Nosocomial infections occur in up to 65% of adult ECMO patients, most commonly bloodstream and surgical site infections, and are associated with increased morbidity and mortality.^1^, ^88^, ^89^ Despite this, routine antimicrobial prophylaxis is not recommended by the Extracorporeal Life Support Organization (ELSO) except in specific high-risk groups, due to the lack of evidence showing benefit in reducing infection or improving outcomes.^88^, ^89^, ^93^

A major challenge in stewardship is the altered antimicrobial PK during ECMO. More than 40% of patients fail to achieve therapeutic concentrations of commonly used antibiotics such as piperacillin and vancomycin.^19^ This variability is compounded by factors such as concomitant renal replacement therapy and the underlying critical illness.^7^, ^19^ As a result, TDM is strongly recommended to guide individualized dosing, and avoid toxicity or treatment failure.^7^, ^19^

Diagnostic uncertainty further complicates stewardship. The systemic inflammatory response induced by ECMO can cause fever, leucocytosis, and elevated biomarkers such as C-reactive protein and procalcitonin, which may not accurately distinguish infection from inflammation.^94^, ^95^, ^96^ This often leads to empirical broad-spectrum antibiotic use in the absence of clear infection, promoting resistance.

Additionally, surveillance cultures, particularly from cannula sites or respiratory specimens, may drive inappropriate treatment of colonization, and the absence of ECMO-specific diagnostic protocols contributes to inconsistent practice.97., 98, 99 Antimicrobial stewardship protocols, including restricted prophylactic use, early de-escalation, and targeted therapy based on microbiology have been shown to reduce overall antimicrobial consumption without increasing infection rates.^93^, ^100^ However, stewardship efforts must balance the risk of under-treatment in a population at high risk for rapid clinical deterioration. In summary, optimal stewardship in ECMO patients requires a tailored, multidisciplinary approach that integrates PK considerations, diagnostic stewardship, and dynamic reassessment.^7^, ^19^, ^88^, ^93^, ^100^ By embedding stewardship principles into ECMO care pathways, institutions can improve patient outcomes, reduce resistance, and ensure that antimicrobial therapy remains both effective and judicious.

As the field evolves, there is a pressing need for ECMO-specific AMS guidance, expanded access to TDM, and robust outcome-linked data to inform precision antimicrobial therapy in this population.

### Future directions

Future directions for understanding antimicrobial PK in ECMO-supported patients demand a cohesive approach to address significant research gaps and improve clinical outcomes.^101^ Current literature is limited by heterogeneity in patient populations, ECMO modalities, and study designs without non-ECMO comparators, often relying on small case series or case reports. This lack of robust, high-quality PK data for commonly used anti-infectives in ECMO complicates meaningful analysis and clear dosing recommendations.

A key gap is the absence of data linking drug concentration measurements to actual patient PD outcomes—namely, infection resolution, toxicity, and survival. Most existing studies focus on drug exposure (such as trough concentrations or area-under-curve values) without connecting them to clinical endpoints. Future research must prioritize trials and observational studies that pair PK sampling with validated PD outcomes, as optimal exposure alone is insufficient if not tied to tangible improvements in patient health.

There is also a pressing need for studies conducted in homogenous patient groups, including a non-ECMO comparator group, with standardized ECMO modalities and establishing consensus on sampling protocols. Variations in factors such as cannulation type, circuit materials, severity of illness, and concurrent therapies confound interpretation of PK data and limit generalizability. Co-ordinated, multicentre research in more uniform populations, using consistent sampling methodologies and timing, would facilitate precise and applicable dosing recommendations.

Complementary approaches have harnessed sequestration data from ex vivo circuits to parameterize physiologically based PK models, enhancing predictive accuracy for adult critically ill patients on ECMO. By combining experimental, preclinical, and computational strategies, future research will be better positioned to deliver evidence-based, individualized antimicrobial dosing recommendations.

## Conclusions

Anti-infective dosing in ECMO-supported patients should employ exposure-optimizing strategies tailored to critical illness, incorporating TDM when available. This should account for complex interactions between patient, device, and drug properties. While emerging data provide guidance, prospective studies focused on clinical outcomes remain essential. Therapeutic drug monitoring is advised when feasible to support individualized therapy.

## Authors’ contributions

The manuscript was initiated by HL. ALD, Y-HL and HL conducted literature review and writing of the original draft. All authors provided critically review of the manuscript and approved the final version of the manuscript.

## Patient consent

This review does not include factors necessitating patient consent.

## Declaration of Competing Interest

The authors declare the following financial interests/personal relationships which may be considered as potential competing interests: All authors declare that they have no known competing financial interests or personal relationships that could have appeared to influence the work reported in this paper.
